# Correlation Between Poor Defecation Habits and Postoperative Hemorrhoid Recurrence

**DOI:** 10.3389/fsurg.2022.930215

**Published:** 2022-06-17

**Authors:** Qing Li, Roshan Ara Ghoorun, Li Li, Heng Zhang, Dan Zhang, Haihua Qian, Dong-Lin Ren, Dan Su

**Affiliations:** ^1^ Department of Anorectal Surgery, The Affiliated Hospital of Nanjing University of Chinese Medicine, Nanjing, China; ^2^ Department of Anorectal Surgery, The Affiliated Xing Tai People Hospital of Hebei Medical University, Xingtai, China; ^3^ Department of Colorectal Surgery, The Sixth Affiliated Hospital (Gastrointestinal & Anal Hospital), Sun Yat-sen University, Guangzhou, China

**Keywords:** defecation habit, obstructive defecation syndrome, hemorrhoid, recurrence, risk factors

## Abstract

**Background:**

The relationship between hemorrhoid recurrence and poor defecation habits is poorly understood. This study aimed to analyze the effects of poor defecation habits on postoperative hemorrhoid recurrence.

**Materials and Method:**

We performed a retrospective study on 1,162 consecutive patients who underwent a surgical procedure for hemorrhoids at the Sixth Affiliated Hospital of Sun Yat-Sen University from December 2016 to May 2020. All patients were followed for 12 months post-operatively. Patients were monitored for disease recurrence. Patient defecation habits were assessed using an obstructive defecation syndrome (ODS) score.

**Results:**

Patients with a score of 0–4 had a mild defecation disorder, 5–8 a moderate defecation disorder, and 9 or more ODS. Of the 1,162 patients, 1,144 (98.45%) had a mild defecation disorder, 13 (1.12%) had a moderate defecation disorder, and 9 (0.43%) had ODS. Older patients were significantly more likely to have worse defecation habits (*P* < 0.001). A higher ODS score correlated with a higher maximum anal squeeze pressure (*P* = 0.07) and a more severe inability for the anus to relax during simulated evacuation (*P* = 0.002). The maximum rectum threshold was also found to be the highest in ODS patients (*P* = 0.010). The proportion of Procedure for prolapsing hemorrhoids (PPH) was the highest in the moderate defecation disorder group (53.85), followed by the ODS group (40.00) and the mild defecation disorder group (*P* = 0.023). Recurrence occurred in 5.51% of patients in the mild defecation disorder group, 38.46% of the moderate defecation disorder group, and 60% of the ODS group (*P* < 0.001). Multivariate analysis confirmed a higher ODS score (*P* < 0.001) was an independent predictor of recurrence. Furthermore, patients who occasionally exercised (*P* = 0.01) and patients who exercised regularly (*P* = 0.021) were less likely to have a recurrence.

**Conclusion:**

Patients with unresolved defecation disorders are more likely to have their hemorrhoids recur and are unlikely to be satisfied with surgical management.

## Introduction

Hemorrhoids are the most common anorectal disease and have a considerable impact on health care expenditure (1). The prevalence of hemorrhoids in the general population is as high as 50% (2), affecting a considerable proportion of adults of all ages and genders (3). Current causes of hemorrhoids include chronic straining during defecation, lack of physical exercise, diarrhea, pregnancy, and inadequate fiber intake (4). There is a close link between hemorrhoids and constipation (5, 6). Hard or large stools and exertion during defecation are associated with an increased prevalence of hemorrhoids. Bowel habit regulation is essential to managing hemorrhoids (7–9). For instance, oral fiber is adjusted for constipation symptoms (5, 10) and flavonoids decrease the risk and recurrence rate of hemorrhoids (11).

Patients diagnosed with obstructive defecation syndrome (ODS) usually have long-standing constipation. The surgical treatment of defecation disorders is still very controversial, with unacceptable effects on patient quality of life (12). Hemorrhoid management is usually conservative, and only 20% of patients require surgical management (13). Similarly, before developing hemorrhoidal symptoms patients usually suffer from difficulty defecating (14). Many patients undergo surgery for this, often without treating the underlying cause of their symptoms. Unsurprisingly, the postoperative recurrence rate of hemorrhoids is still high (15, 16), with multiple studies attributing recurrence to surgical technique (17, 18).

Although surgical technique is widely accepted as the main reason for hemorrhoid treatment failure, the relationship between poor defecation habits and hemorrhoid recurrence is poorly understood. This study aimed to analyze the effects of poor defecation habits on postoperative hemorrhoid recurrence. We hypothesized that patients with more severe defecation disorders would be more likely to see their hemorrhoids recur following surgery. Patients with a severe defecation disorder are less likely to be satisfied with their surgical outcome regardless of the method used to perform the surgery.

## Materials and Methods

### Study Design and Participant Selection

**Figure 1:**
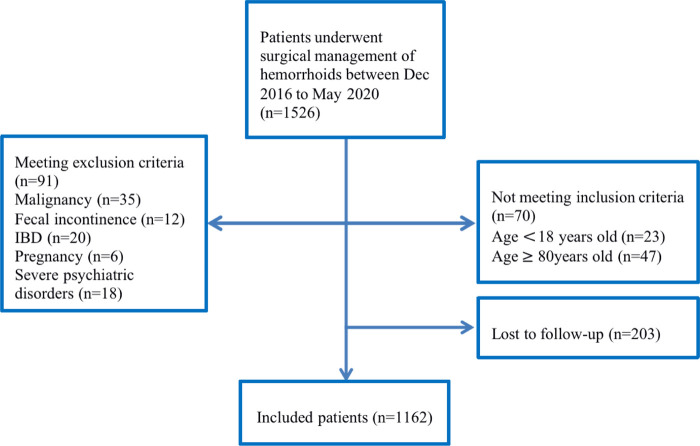
Flow diagram study sample selection.

The unit protocol and study format were approved by the Sixth Affiliated Hospital of Sun Yat-Sen University (Approval code: 2022ZSLYEC-096). This retrospective study was performed on 1,162 consecutive patients who underwent a surgical procedure for hemorrhoids from December 2016 to May 2020 (Figure 1). All patients were followed for 12 months post-operatively. Inclusion criteria were age > 18 and ≤80 years old, a clinical diagnosis of hemorrhoids, and surgery performed by a surgeon with at least 5 years of experience with all three common surgical techniques. Exclusion criteria were previous hemorrhoidal surgical management, malignancies, fecal incontinence, pregnancy, and severe psychiatric disorders.

### Preoperative Preparation, Surgical Methods

All patients underwent routine pre-operative bloodwork, electrocardiograms, and chest X-rays to rule out surgical contraindications. They also underwent a pre-operative colonoscopy to exclude more serious diseases such as inflammatory bowel disease, proliferative polyps, and colorectal cancer. All patients underwent pre-operative anorectal manometry, which was preceded by a phosphate enema. Anorectal manometry was performed with patients in the left lateral position with their hips flexed. Anorectal manometric parameters such as anorectal pressure, rectal sensation, and neural reflexes were recorded. All patients received a pre-operative bowel enema the night before surgery, and a prophylactic antibiotic was injected 30 min before the surgical procedure was started. Most patients had spinal anesthesia and were placed in the jackknife or left lateral position. Tape was attached to both sides of the buttocks to expose the anus.Some other patients underwent local anesthesia, because these patients were able to tolerate the relatively simple procedure under local anesthesia. General anesthesia was performed in some patients because of lumbar lesions, coagulopathy, or psychosomatic disorders.

### Surgical Modalities

Many patients had a combination of surgical therapies. The Goligher classification is the most widely used classification for HD. Surgical management was performed based on the Goligher Classification of hemorrhoids (19), the type of health insurance patient’s choice. Patients with bleeding hemorrhoids underwent either Hemorrhoid injection sclerotherapy (IS)or rubber band ligation (RBL), which was performed either in the treatment room using 1% lidocaine as a local anesthetic or in the operation theater under spinal anesthesia. IS and RBL were used for the treatment of I, II, and III-degree HD. A recent multicentre study showed that Sclerotherapy with 3% polidocanol foam is a safe, effective, painless, repeatable and low-cost procedure in patients with bleeding hemorrhoids, especially in the treatment of 2nd-degree hemorrhoids (20). After a thorough digital rectal examination (DRE), an anoscope was inserted to fully expose the hemorrhoids. Sclerotherapy was mainly performed if the patient suffered from hemorrhoidal bleeding. It was done using “Shaobei” (Taifeng, Henan, China) and lidocaine in a 1:1 ratio. The mixture was injected into the submucosa of each hemorrhoid tissue, paying attention to avoid any blood vessels. The total injection volume was generally less than 20 mL. Rubber band ligation was used on patients with bleeding hemorrhoids. It was performed using a ligation device to ligate the hemorrhoid, bleeding point, or tissue superior to the hemorrhoid. There were generally less than four ligation sites. Procedure for prolapsing hemorrhoids (PPH) and the Milligan-Morgan hemorrhoidectomy (MMH) was indication for symptomatic III- and IV-degree HD. Preoperative clinical evaluation is essential for HD patient treatment and outcome and classification systems are useful for the therapeutic choice (21). Our patients with obvious protrusions underwent PPH or MMH, which were usually performed under spinal anesthesia. Endotracheal anesthesia was used in patients who had any contraindications to spinal anesthesia, such as a coagulopathy. The patient was usually in the prone jackknife position or the lithotomy position during surgery. After a thorough DRE and insertion of an anal speculum, the surgeon identified the hemorrhoids that were to be excised. PPH was performed as previously described (22). For MMH, tissue forceps were used to grasp and lift the external hemorrhoid towards the surgeon at the mucocutaneous junction. Monopolar electrocautery or a pair of scissors were used to make a V-shaped incision in the skin around the base of hemorrhoid. The dissection continued into the submucosal space to peel the entire hemorrhoid from its bed. The pedicle was ligated, and the distal part of the hemorrhoid was excised. All of the other hemorrhoids were similarly treated, leaving a skin bridge in-between to avoid stenosis. Large wounds were closed and sutured with 3-0 absorbable thread for aesthetic reasons. An absorbable sponge dressing was placed in the anal canal when the procedure was completed. Post-operatively, the patient was advised to take an adequate amount of dietary fiber and water.

### Follow-Up

All cases were followed for at least 12 months from the date of surgery for any disease recurrence. Recurrence was defined as unresolved bleeding or prolapse at the site where surgical management was performed. It is difficult for patients to accurately describe their defecation habits. Given the anatomic, functional, and sometimes psychological factors involved in defecation there may be a variety of clinical presentations of ODS. We therefore used the ODS severity index to evaluate patients’ defecation habits (12). The ODS score (23, 24) was chosen to assess these patients’ defecation habits (Table 1). A score of 0–4 was classified as a mild defecation disorder, 5–8 as a moderate defecation disorder, and 9 or more as ODS.

**Table 1:** Obstructed defecation score.

Frequency of defecation	
1–2 defecations every 1–2 days	0
2 defecations/week or 3 defecations or attempts/day	1
1 defecation/week or 4 defecations or attempts/day	2
<1 defecation/week or >4 defecations or attempts/day	3
Intensity of straining	
No or light	0
Moderate	1
Intense	2
Duration of straining	
Short	1
Prolonged or many times	2
Incomplete evacuation	
Never	0
≤1 time/week	1
2 times/week	2
>2 times/week	3
Rectoperineal discomfort	
Never	0
≤1 time/week	1
2 times/week	2
>2 times/week	3
Reduction of activities	
None	0
<25%	2
25%–50%	4
>50%	6
Laxatives	
Never	0
<25% of defecations	1
25%–50% of defecations	3
>50% of defecations	5
Always	7
Enemas	
Never	0
<25% of defecations	1
25%–50% of defecations	3
>50% of defecations	5
Always	7
Digitation	
Never	0
<25% of defecations	1
25%–50% of defecations	3
>50% of defecations	5
Always	7

### Statistical Methods

The statistical analyses were performed using SPSS 26.0 (Armonk, NY, USA). All tests were two-way, and *P* < 0.05 was considered statistically significant. Mean ± standard deviation was used to describe continuous variables, frequency and frequency were used to describe categorical variables, and the analysis of variance or chi-square tests were used to identify statistically significant differences between groups. Univariate logistic regressions were used to identify correlations between various factors and hemorrhoid recurrence. The univariate results were included in a multivariate logistic regression model for further analysis, and stepwise regression was used to screen variables.

## Results

### Patient Demographics

A total of 1,162 consecutive patients underwent a hemorrhoidectomy at our institution and followed up for at least 12 months. Of the 1,162 patients, 1,144 (98.45%) had a mild defecation disorder, 13 (1.12%) had a moderate defecation disorder and 9 (0.43%) had ODS. Older age was significantly correlated with worsen defecation habits (*P* < 0.001) (Table 2).

**Table 2:** Patient demographics.

Variable	Mild defecation disorder	Moderate defecation disorder	ODS	Statistic	*P*
Number of cases	1144 (98.45)	13 (1.12)	5 (0.43)	–	–
Gender					
Male	533 (46.59)	6 (46.15)	1 (20.00)	1.303	0.630
Female	611 (53.41)	7 (53.85)	4 (80.00)		
Age (years)	40.91 ± 12.25	56.92 ± 17.58	58.20 ± 24.34	15.510	**<0.001**
Height (m)	1.65 ± 0.08	1.65 ± 0.07	1.58 ± 0.06	1.784	0.168
Weight (kg)	51.05 ± 11.32	57.23 ± 8.50	55.30 ± 6.20	1.374	0.253
BMI (kg/m^2^)	22.46 ± 3.38	21.05 ± 3.05	22.33 ± 3.46	1.115	0.328
Smoking habit					
No	963 (84.18)	9 (69.23)	5 (100.00)	2.586	0.251
Yes	181 (15.82)	4 (30.77)	0 (0.00)		
Alcohol consumption					
Rarely	1005 (87.85)	13 (100.00)	5 (100.00)	1.250	0.494
Often	139 (12.15)	0 (0.00)	0 (0.00)		
Physical activity					
Rarely	375 (32.78)	4 (30.77)	2 (40.00)	3.823	0.408
Seldom	555 (48.51)	4 (30.77)	2 (40.00)		
Regular exercise	214 (18.71)	5 (38.46)	1 (20.00)		
Average daily working hours					
<5 h	226 (19.45)	4 (30.77)	2 (40.00)	2.916	0.554
5–8 h	610 (53.32)	7 (53.85)	2 (40.00)		
>8 h	308 (26.92)	2 (15.38)	1 (20.00)		
Taste preference					
Not partial	487 (42.57)	3 (76.92)	0 (0.00)	5.348	0.054
Partial addiction	657 (57.43)	10 (23.08)	5 (100.00)		
Amount of drinking water					
≤2,000 mL/day	566 (49.48)	6 (46.15)	3 (60.00)	0.377	0.933
>2,000 mL/day	578 (50.52)	7 (53.85)	2 (40.00)		
Dietary condition					
Low fiber diet	765 (66.87)	8 (61.54)	4 (80.00)	0.540	0.847
High fiber diet	379 (33.13)	5 (38.46)	1 (20.00)		

*The bold values has Statistically significant P value.*

### Pre-Operative Anorectal Manometry

We aimed to understand the effects of poor defecation habits on pre-operative anorectal function (Table 3). A higher ODS score correlated with a higher maximum anal squeeze pressure (*P* = 0.07) and a more severe inability for the anus to relax during simulated evacuation (*P* = 0.002). ODS patients also had the highest maximum rectum threshold (*P* = 0.010).

**Table 3:** Physiological and biochemical indices.

Variable	Mild defecation disorder	Moderate defecation disorder	ODS	F	*P*
Albumin g/L	43.46 ± 5.72	42.97 ± 2.71	42.68 ± 2.11	0.091	0.913
Hemoglobin g/L	128.67 ± 25.59	126.32 ± 24.75	111.90 ± 19.13	1.123	0.326
Platelets (109L)	248.97 ± 66.55	226.71 ± 63.46	231.52 ± 107.71	0.882	0.414
Mean anal resting pressure (MERP) (mmHg)	90.97 ± 24.49	82.10 ± 14.79	108.28 ± 49.36	2.092	0.124
Length of the anal canal (cm)	3.61 ± 2.26	3.70 ± 0.33	3.44 ± 0.60	0.025	0.975
Maximum anal squeeze pressure (MSP) (mmHg)	210.08 ± 63.01	240.75 ± 94.91	285.60 ± 78.08	4.991	**0.007**
Anal relaxation rate (ARR) during simulated evacuation (%)	28.09 ± 17.40	25.92 ± 17.82	0.20 ± 0.18	6.505	**0.002**
First sensation (FST) (mL)	39.62 ± 15.21	44.62 ± 12.66	50.00 ± 10.00	1.850	0.158
First defecation threshold (mL)	57.87 ± 21.28	60.77 ± 18.47	68.00 ± 21.68	0.682	0.506
Maximum tolerable threshold of the rectum (mL)	115.61 ± 31.53	110.77 ± 26.91	158.00 ± 47.65	4.656	**0.010**

*The bold values has Statistically significant P value.*

### Goligher Classification and Surgical treatment

1 RBL procedure and 11 IS procedures were done spinal anesthesia due to lumbar lesions, coagulopathy, or psychosomatic disorders. 5 MMH procedures were performed under local anesthesia because these patients were able to tolerate the relatively simple procedure under local anesthesia. Endotracheal intubation was performed in 12 combined PPH and MMH procedures, 9 combined MMH and RBL procedures, and 5 MMH procedure because of lumbar lesions, coagulopathy, or psychosomatic disorders. The other 1,119 patients routinely received spinal anesthesia.

Most of the low defecation disorder patients underwent an MMH procedure (77.54%) while sclerotherapy was least utilized (7.97%). Only the use of PPH was statistically significant between groups. The proportion of PPH was the highest in the moderate defecation disorder group (53.85%), followed by the ODS group (40.00%) and the mild defecation disorder group (*P* = 0.023). The proportion of single operation and combined operation was 185 (15.92%) and 977 (84.08%) respectively. The proportion of single operation and compound operation in each group of defecation disorder was almost the same. (*P *= 0.876). Results are shown in Table 4. There was no statistical significance observed on the use of single or combined surgeries (*P* = 1.000). No statistical significance was observed among the four surgical methods _ PPH (*P* = 0.943), MMH (*P* = 0.649), RBL (*P* = 0.499), IS (*P* = 0.332). There was also no statistical significance observed between Goligher grade and recurrence rates (*P *= 0.944) (Table 5).

**Table 4:** Correlation between surgical modality and defecation habits.

Surgical modality	Total number of cases	Mild defecation disorder	Moderate defecation disorder	ODS	*χ^2^*	*P*
PPH						
No	896 (77.11)	887 (77.53)	6 (42.15)	3 (60.00)	7.442	0.023
Yes	266 (22.89)	257 (22.47)	7 (53.85)	2 (40.00)		
M-M						
No	261 (22.46)	257 (22.46)	3 (23.08)	1 (20.00)	0.185	1.000
Yes	901 (77.54)	887 (77.54)	10 (76.92)	4 (80.00)		
RBL						
No	391 (33.65)	381 (33.30)	8 (61.54)	2 (40.00)	4.626	0.085
Yes	771 (66.35)	763 (66.70)	5 (38.46)	3 (60.00)		
IS						
No	1081 (93.03)	1064 (93.01)	12 (92.31)	5 (100.00)	0.279	0.730
Yes	81 (7.97)	80 (6.99)	1 (7.69)	0 (0.00)		
Surgical modality						
Only one	185 (15.92)	182 (15.91)	2 (15.38)	1 (20.00)	#	0.876
Combined	977 (84.08)	962 (84.09)	11 (84.62)	4 (80.00)		

**Table 5:** Effects of surgical modality and goligher grade on hemorrhoid recurrence rates.

	All	No Recurrence	Recurrence	*P*
	*N* = 1162	*N* = 1091	*N* = 71	
PPH				0.943
No	896 (77.11%)	842 (77.18%)	54 (76.06%)	
Yes	266 (22.89%)	249 (22.82%)	17 (23.94%)	
M-M				0.649
No	261 (22.46%)	243 (22.27%)	18 (25.35%)	
Yes	901 (77.54%)	848 (77.73%)	53 (74.65%)	
RBL				0.499
No	391 (33.65%)	364 (33.36%)	27 (38.03%)	
Yes	771 (66.35%)	727 (66.64%)	44 (61.97%)	
IS				0.332
No	1081 (93.03%)	1017 (93.22%)	64 (90.14%)	
Yes	81 (6.97%)	74 (6.78%)	7 (9.86%)	
Surgical modality				1.000
Only one	185 (15.92%)	174 (15.95%)	11 (15.49%)	
Combined	977 (84.08%)	917 (84.05%)	60 (84.51%)	
Goligher grade				0.944
1	42 (3.61%)	39 (3.57%)	3 (4.23%)	
2	251 (21.60%)	237 (21.72%)	14 (19.72%)	
3	675 (58.09%)	633 (58.02%)	42 (59.15%)	
4	194 (16.70%)	182 (16.68%)	12 (16.90%)	

### Unresolved Poor Defecation Habits and Hemorrhoids Recurrence

We assessed the impact of an unresolved defecation habit on hemorrhoidal recurrence using linear regression (Table 6). Recurrence occurred in 5.51% of the mild defecation disorder group, 38.46% of the moderate defecation disorder group, and 60% of the ODS group (*P* < 0.001).

**Table 6:** Effects of poor defecation habits on hemorrhoid recurrence.

Group	Recurrence	*β*	Standard Error	Wald	*P*	OR (95% CI)	*P*-trend
Mild defecation disorder	63 (5.51)	–	–	–	–	1.00	**<0.001**
Moderate defecation disorder	5 (38.46)	2.373	0.585	16.468	**<0.001**	10.72 (3.41, 33.73)	
ODS	3 (60.00)	3.248	0.922	12.409	**<0.001**	25.74 (4.22, 156.82)	

*The bold values has Statistically significant P value.*

### Analysis of Factors Influencing Hemorrhoids Recurrence

We aimed to identify factors associated with hemorrhoid recurrence. In a univariate analysis (Table 7), ODS score (*P* < 0.001), physical activity (*P* = 0.001), and BMI (*P* = 0.014)) were significantly associated with hemorrhoid recurrence. There was no statistical significance observed between recurrence and surgical modality (*P* = 0.919). Multivariate analysis (Table 8) confirmed that ODS score (OR = 1.380, *P* < 0.001) was independent risk factor for hemorrhoid recurrence. Every increment in the ODS score resulted in an increased risk of hemorrhoid recurrence of 1.38 times. Interestingly, the recurrence risk of patients who exercised occasionally was 0.445 and that of for those who exercised regularly was 0.337, implying that physical activity has a protective effect against hemorrhoid recurrence.

**Table 7:** Univariate analysis for hemorrhoid recurrence.

Variable	*β*	Standard Error	Wald	*P*	OR (95%CI)
ODS score	0.431	0.073	5.873	**<0** **.** **001**	1.539 (1.338, 1.787)
Gender					
Female	–	–	–	–	1
Male	−0.369	0.252	−1.465	0.143	0.691 (0.417, 1.125)
Age (years)	−0.008	0.01	−0.759	0.448	0.992 (0.972, 1.012)
BMI (kg/m^2^)	−0.102	0.042	−2.453	**0** **.** **014**	0.903 (0.83, 0.977)
Smoker					
No	–	–	–	–	1
Yes	0.104	0.328	0.318	0.751	1.11 (0.558, 2.037)
Alcohol consumption					
No	–	–	–	–	1
Yes	0.071	0.369	0.191	0.848	1.073 (0.488, 2.105)
Physical activity					
Rarely	–	–	–	–	1
Seldom	−0.925	0.268	−3.446	**0** **.** **001**	0.397 (0.231, 0.666)
Often	−1.075	0.398	−2.702	**0** **.** **007**	0.341 (0.146, 0.707)
Daily sitting habit					
<5h	–	–	–-	–	1
5–8h	0.616	0.396	1.556	0.12	1.852 (0.897, 4.328)
>8h	0.815	0.418	1.951	0.051	2.26 (1.038, 5.461)
Taste preference					
Partial	–	–	–	–	1
Specific	−0.298	0.274	−1.089	0.276	0.742 (0.441, 1.296)
Water consumption					
≤2,000 mL/day	–	–	–	–	1
>2,000 mL/day	0.2	0.246	0.811	0.417	1.221 (0.755, 1.988)
Diet					
Low fiber	–	–	–	–	1
High fiber	−0.228	0.25	−0.913	0.361	0.796 (0.49, 1.31)
Mean resting pressure of anal sphincter (mmHg)	0.003	0.005	0.532	0.594	1.003 (0.993, 1.012)
High-pressure zone (cm)	−0.072	0.092	−0.775	0.438	0.931 (0.725, 1.057)
Maximum anal sphincter pressure (mmhg)	0.001	0.002	0.774	0.439	1.001 (0.998, 1.005)
Anal relaxation rate (%)	−0.009	0.006	−1.685	0.092	0.991 (0.98, 1.002)
Initial sensory threshold (mL)	0.003	0.008	0.457	0.648	1.003 (0.988, 1.018)
Initial defecation threshold (mL)	0.009	0.005	1.827	0.068	1.009 (0.999, 1.018)
Maximum tolerance threshold (mL)	0	0.004	0	1	1.00 (0.992, 1.007)
Duration course (Months)	0.001	0.002	0.538	0.59	1.001 (0.998, 1.004)
Goligher grade					
1					1
2	−0.264		0.659	0.689	0.768 (0.237–3.44)
3	−0.148		0.620	0.812	0.863 (0.296–3.669)
4	−0.154		0.669	0.818	0.857 (0.257–3.89)
Surgical modality					
Only one	–	–	–	–	1
Combined	0.034	0.338	0.102	0.919	1.035 (0.554, 2.114)
PPH					
No	–	–	–	–	1
Yes	0.063	0.287	0.218	0.828	1.065 (0.59, 1.831)
MMH					
No	–	–	–	–	1
Yes	−0.17	0.282	−0.602	0.547	0.844 (0.494, 1.505)
RBL					
No	–	–	–	–	1
Yes	−0.203	0.253	−0.805	0.421	0.816 (0.50, 1.354)
IS					
No	–	–	–	–	1
Yes	0.408	0.416	0.98	0.327	1.503 (0.61, 3.188)

*The bold values has Statistically significant P value.*

**Table 8:** Multivariate analysis of hemorrhoid recurrence.

Variable	*β*	Standard Error	Wald	*P*	OR(95% CI)
ODS score	0.322	0.089	3.605	**<0.001**	1.380 (1.157, 1.651)
BMI (kg/m^2^)	−0.037	0.049	−0.762	0.446	0.963 (0.872, 1.054)
Physical activity					
Rarely	–	–	–	–	1.00
Seldom	−0.809	0.312	−2.592	**0.01**	0.445 (0.239, 0.817)
Often	−1.088	0.473	−2.302	**0.021**	0.337 (0.126, 0.814)

*The bold values has Statistically significant P value.*

## Discussion

The primary aim of this study was to understand if patients with unresolved poor defecation habits are at a higher risk of recurrence following hemorrhoidectomy. Our results show 1,144 (98.45%) had a mild defecation disorder, 13 (1.12%) had a moderate defecation disorder, and 9 (0.43%) had ODS. Older patients were significantly more likely to have worse defecation habits (*P* < 0.001). A higher ODS score correlated with a higher maximum anal squeeze pressure (*P* = 0.07) and a more severe inability for the anus to relax during simulated evacuation (*P* = 0.002). The maximum rectum threshold was also found to be the highest in ODS patients (*P* = 0.010). The proportion of (PPH) was the highest in the moderate defecation disorder group (53.85), followed by the ODS group (40.00) and the mild defecation disorder group (*P* = 0.023). Recurrence occurred in 5.51% of patients in the mild defecation disorder group, 38.46% of the moderate defecation disorder group, and 60% of the ODS group (*P* < 0.001). Multivariate analysis confirmed a higher ODS score (*P* < 0.001) was an independent predictor of recurrence. Furthermore, patients who occasionally exercised (*P* = 0.01) and patients who exercised regularly (*P* = 0.021) were less likely to have a recurrence.

The pathophysiology of hemorrhoids is not fully understood. A recent genome-wide association study (GWAS) analysis of 944,133 individuals found that hemorrhoids, which is a partially inherited disease, could be affected by functional gastrointestinal diseases (FGID) through genotype-driven modulation of Caja interstitial cell (ICC) function (25). ICCs are present throughout the human gastrointestinal tract and help in normal intestinal function and peristalsis. Few studies have shown differences in ICC distribution between ODS and non-ODS patients (26, 27), implying that patients with FGID are more likely to suffer from hemorrhoids.

It is difficult for patients to accurately describe their defecation habits. Given the anatomic, functional, and sometimes psychological factors involved in defecation there may be a variety of clinical presentations of ODS. It is therefore recommended that the ODS severity index is used evaluate patient defecation habits (12). ODS score describes a series of complex symptoms such as repeated straining, difficulty evacuating, using laxatives or an enema to defecate, using digital means, spending an excessive amount of time on the toilet during defecation, feelings of incomplete evacuation, perineal pain, and rectal discomfort (23, 24). Our results show that the defecation disorder score is an independent predictor for hemorrhoidal recurrence. Every one-point increment increased the risk of hemorrhoid recurrence by 1.38 times (*P* = <0.001). Furthermore, risk of hemorrhoid recurrence in the moderate defecation disorder group was 10.72 times higher, and 25.74 higher in the ODS group (*P* < 0.001). According to previous literature reports, key components of the pathogenesis and aggravation of hemorrhoids are rectal mucosal prolapse and increased anal pressure (28). Defecation disorder and pelvic floor disease cause the disintegration of the muscle fibers of Treitz (29). Straining during defecation may lead to excessive intra-abdominal pressure, rendering defecation ineffective and therefore multiple evacuations are required. Straining also impairs venous drainage of the hemorrhoids (30). Avoiding forced defecation has been shown to limit the prolapse of hemorrhoids (31).

We also found that physical activity has a protective effect against hemorrhoidal recurrence. Patients who exercised occasionally (*P* = 0.01) or regularly (*P* = 0.021) were less likely to have a recurrence. Mild physical activity can accelerate gastrointestinal transit and increase the stimulation of abdominal muscles, therefore aiding in the movement of stool into the rectum (32). However, high-intensity exercises can often lead to gastrointestinal distress when associated with either dehydration or increased intra-abdominal pressure (33). As noted by Marco et al. (5), we recommend that hemorrhoidal patients abandon a sedentary lifestyle and practice mild exercises such as walking, swimming, and yoga. This approach can result in improved circulation, strengthen the pelvic floor muscles, improve anorectal function, and prevent a defecation disorder.

We also found that older patients were more likely to have worse defecation habits (*P* < 0.001). The physiologic changes that result from poor defecation habits are not fully understood (34). Aging is associated with a higher prevalence of constipation (35) and impaired collagen quality. The ratio of connective tissue to muscle tissue increases with age and may play a role in the development of rectal mucosal or rectal prolapse in the elderly (36). This imbalance would result in the inability of the muscles to adequately contract to support the internal hemorrhoidal plexus (37). These factors would lead to sliding of the anal cushion, relaxation of the cushion’s connective tissue, and reduced venous return to the middle rectal vein and the superior rectal vein (38). A recent study (39) by Stanford Medical School showed that elderly patients had more severe prolapse symptoms than younger patients. With respect to anorectal function, we found that a higher ODS score was associated with a higher maximum anal squeeze pressure (*P* = 0.07), a more severe inability for the anus to relax during simulated evacuation (*P* = 0.002), and a higher maximum rectum threshold (*P* = 0.010). Increased maximum anal resting pressure, (40) increased internal anal sphincter activity, and a higher maximum rectum threshold (41) are common in patients with hemorrhoids and ODS.

Our results show a hemorrhoid recurrence rate of 6.11% following surgery, which is consistent with recurrence rates of 2% to 8% cited by previous clinical studies (42–44). There was no significant difference in recurrence rate based on the surgical procedure performed, which is consistent with the review by Naldini et al. (45). Patients with a moderate defecation disorder or ODS were more likely to undergo PPH. This is because these patients were more likely to have a higher degree of protrusion compared with mild defecation disorder patients.

Our findings are in line with those of a recent systematic review that reported that functional evacuation disorder, dyssynergic defecation, and abnormal balloon expulsion were more frequent in patients with hemorrhoids compared with healthy subjects (*P* < 0.0001) (46). The review concluded that it would be more effective to treat functional constipation instead of repeating RBL procedures. Pelvic floor physiotherapy was also deemed necessary to improving the long-term results of hemorrhoid treatment (14). We believe that it is necessary to evaluate the bowel habits of hemorrhoidal patients prior to surgery. We recommend performing preoperative anorectal manometry if the ODS score is 5 or more. Patients with defective anorectal function should be treated preoperatively conservatively or through biofeedback (47, 48). Patients should also be regularly followed for their symptoms. If poor defecation habits persist, defecation habit scores should be re-evaluated. Once these habits are improved, surgical management can be considered.

This study has several limitations. First, this is a retrospective study that has a small sample size of patients with a moderate defecation disorder or ODS. Prospective randomized controlled clinical trials need to be performed in the future. Secondly, we did not investigate whether there is a causal relationship or co-disease mechanism between ODS and hemorrhoids. Neural pathways and the role of the anal sphincter and pelvic floor muscles during defecation needs to be further studied. Further basic research projects on genetic and molecular mechanisms will be considered in future work.

## Conclusion

The defecation habits of patients with hemorrhoids should be scored both perioperatively and postoperatively. They should be encouraged to normalize their defecation habit and pick the right exercise routine. A scoring system will help guide the preoperative conversation, analyze the patient’s prognosis, managing the patient’s post-operative expectations, and reduce hemorrhoid recurrence.

## Data Availability

The original contributions presented in the study are included in the article/Supplementary Material, further inquiries can be directed to the corresponding author/s.
